# The importance of asking questions and doing things for a reason

**DOI:** 10.21470/1678-9741-2021-0950

**Published:** 2021

**Authors:** Rodolfo A. Neirotti

**Affiliations:** 1 Master in Public Administration, Harvard Kennedy School. Harvard University, Cambridge, Massachusetts, United States.; 2 Clinical Professor of Surgery and Pediatrics, Emeritus Michigan State University, Michigan, United States.

NB: Some of my thoughts expressed here have been shared in previous presentations.

Arts, artists and intellectuals, with their capacity of seeing with new eyes, sensing and perceiving mastery, finding beauty, meaning, elegance, rhythm, melody, harmony and composition-can help us to understand many aspects of our life, like:

"The two most important days in your life are the day you are born and the day you find out why."

Mark Twain. 1835-1910.

"Whoever has a reason to live will almost always find how." Frederick Nietzsche

"I keep six honest serving-men. They taught me all I knew. Their names are: WHY, HOW, WHAT, WHEN, WHERE, WHO."

An exercise used as a problem analysis method based on Rudyard Kipling's poem, to ensure that all aspects were covered to improve interactions.\

*"Stay away from difficult people-*they have a problem for every solution. Attributed to Albert Einstein. *How to handle them?* This is a common question of business people that encloses the assumption that they are dealing with abrasive, competitive, and unethical behavior that makes them think that they are right and the other party wrong. However, in a conflictive situation the other party can think that the other is difficult and obstinate!

*Daily we ask many questions, WHY? WHAT? HOW?* Why we do things in difficult contexts due to cultural, political and economic adversity? To learn, pay attention to details: every aspect of your work, adopt an analytic mindset and ask *Why? How?* This Should be based on knowledge instead of feelings, habits and impulses. *What?* We all know what we do for a living-right or wrong.

*Questions are useful tools,* they open lines of communications; give us information; improve interactions, facilitate analysis and diagnostics of a situation; allow us to propose our own ideas; help to understand the priorities of others; stimulate motivation to learn; motivate creativity and more importantly scientific research, explanations and its applications happen in part through questions and answers.

*Questioning everything* helps to understand the world round us and *Why* we do what we do? How do we do that is about technical issues and *What* we do is usually known. *Why* reflects beliefs, reasons, purpose and objectives of an institution that eventually motivate its members to adopt them. *Caution*, failures of human psychology can induce people to consider a single factor. Responsible answers to questions can help to improve the quality of What we do. In addition, observations combined with curiosity and questions help us to learn WHY we do things. (3) *Modified from Marilee Adams; Berrett Koehler Publishers, San Francisco, CA. 2016.*

In general, people and societies know *what* they do and some are aware of *How* things are done but many of them ignore *why* they do *what* they do; that, in the end, has an impact on the outcomes. Please, if you do not know *Why* you do it, do not do it!

*WHY Matters*? *Because it is the question that every project team member should answer* to explain the reason they are pursuing a venture. A compelling *"WHY statement"* is a useful tool that aligns the efforts of the leaders, and team members, to improve the chances of success. It sounds simple, but it's not. Often, a good why requires work and debate.

In medicine, doing things without knowing *Why* is risky. Many of the things that doctors and nurses do continue simply because that is the way we have always done it. Still, if they do know why, it does not mean that it was done correctly! Unfortunately, it can also be due to lack of knowledge, attitude, or practices that eventually became automatic. Doing things for no reason-ignoring why- can involuntary harm institutions and patients. Abraar Karam. BMJ opinion January 17, 2019.

For doing things for a reason, start with *Why* because virtually, everything we do and think is generated by questions *that make you think.* Many projects fail because their members are functioning without a good reason for doing things. Failures are often due to not discussing, agreeing, or learning why workers do things*. K.A. Brown, N.L. Hyer and R. Ettenson. MIT Sloan Management Review. Fall 2013, Vol. 55 NO 1.*

*Why do we work?* It is an appeal to reasoning rather than to emotions. Generally speaking, we work for many reasons. We work to live and live to work. Therefore, understanding why we work should help to improve our attitude, motivation, efficiency, productivity, team work and quality of life.

*How to reconcile with work?* Start by thinking what you owe to work rather than what work owes to you. Will more money motivate us to work harder? Actually, not quite. The reasons people work hard are more fundamental than most realize. Simply, feeling like you are part of the team and made progress on a task can give you the boost you need to keep going. Work for it! *Modified from O. Kazhan, P Rosenfeld. The Atlantic Nov. 06, 2015.*

*WHY education?* Because the educational system does not fulfill its purposes. Therefore, new teaching and learning strategies are needed in this evolving, technologically saturated world. This doesn't mean teaching people to accept a set of beliefs without making a proper analysis! This is because education reduces inequality, social problems, improves quality of life - that includes physical and mental health, family, work, income and the environment. Furthermore, democracy doesn't work without people capable to elect honest and competent politicians. In a country with significant and increasing inequality that is divided by political ideas, religious belief, self-interest, income, and with a heterogeneous population, it does not come as a surprise that education is divided. Social scientists offer competing models of class structure, and most agree that society is stratified, among other factors, by educational attainment. Inequality, poverty, suboptimal education and inadequate health care are barriers to Maslow' Sets of Needs. *American Psychologist Abraham Maslow's Hierarchy of Needs Theory. 1943.* In addition, "No society can surely be flourishing and happy, if the greater part of the numbers is poor and miserable" was the awkward phrase dropped by moral philosopher Adam Smith when he revised his thoughts for "*The Wealth of Nations"* published in 1776.

Altogether, this indicates that there is a need to modernize teaching techniques because there is a gap between what education systems provide and what is currently required by the society and employers. New technologies, have altered people's work and lives, pressing reformers to say that the traditional curriculum is not adequate.

Education and jobs can heal society. Authorities need to identify what skills are necessary for students to succeed in careers and personal lives, and then modernize their curriculums. Asking teachers to focus on a list of poorly defined skills is not enough. Interestingly, Angela Merkel during her address at the 2019 Harvard Graduation, address, stated "Nothing Has to Stay the Way It Is." Because the Berlin Wall limited peoples' opportunities, the German Chancellor invited the crowd to think with imagination about the possibility of precipitating what was previously an unimaginable change. "The Berlin Wall limited my opportunities but it couldn't impose limits on my inner thoughts... and that anything that seems to be set in stone or inalterable can, indeed, change."

Factors that can have either a positive or negative impact on the benefits of asking questions and doing things for a reason:

- *Attitude* is the tendency to respond positively or negatively to work, ideas, persons, objects or situations. In addition, it also impacts the individual's selection of actions, responses to challenges, incentives and prizes. An optimistic attitude, avoids negative thinking, and helps with daily activities. Talent is natural, and attitude cannot be taught.
- *Motivation:* the enthusiasm to do things, and a reason for people's actions, desires and needs. Inculcating motivation is not easy, but it's essential if you want your team to grow and stay satisfied with their jobs.
- *Complacency and false urgency: Complacency*: people do little or nothing to grow and improve, justify why they cannot do, and are unaware of the self-damage. False urgency: when people act and look busy, without adding value to what they are currently doing.
- *Mental laziness* -- a reluctance to doing something despite having the capability-due to a difficulty to put their brains to work. No matter how hard you work to get something done, mental laziness is when you stop midway because it is not easy. It is when you give up because you are tired or you feel that have done enough.
- *Perfectionist*, someone who avoid errors on a personal crusade for flawlessness. A boss, colleague, or even a work friend whose values have almost nothing to do with reality. Studies have tended to focus on their output rather than the effect *they* might have on their team climate or interpersonal relationships.

Finally, if we know *Why*, think carefully about *What* we must preserve. *What* we must improve? And what we must transform? In order to progress.

